# Endoscopic Retrograde Cholangiopancreatography Induced Pancreatic Ascites

**DOI:** 10.7759/cureus.9851

**Published:** 2020-08-18

**Authors:** Jennifer C Asotibe, Ikechukwu Achebe, Olukayode A Busari, Emmanuel Akuna, Hafeez Shaka

**Affiliations:** 1 Internal Medicine, John H. Stroger, Jr. Hospital of Cook County, Chicago, USA; 2 Internal Medicine, NYC Health + Hospitals/Coney Island, New York, USA

**Keywords:** endoscopy ercp, pancreatic ascites, mrcp

## Abstract

Pancreatic pathology is one of the causes of abdominal ascites. The estimated prevalence of pancreatic ascites is 3.5% in patients with chronic pancreatitis and it is mostly caused by pancreatic duct dehiscence in the setting of chronic pancreatitis. Other etiologies include pancreatic pseudocysts, trauma, severe acute pancreatitis and rupture to the pancreas. Management of this condition includes conservative management like holding feeds, total parenteral nutrition, administering somatostatin analogues or sometimes invasive procedures like endoscopic retrograde cholangiopancreatography (ERCP) and surgery. ERCP is an unusual cause of pancreatic ascites and only one other case report has linked an association between ERCP and the development of pancreatic ascites. Our case report contributes to this literature and aims to shed light on this under-reported cause of pancreatic ascites.

## Introduction

Pancreatic ascites typically occurs as a consequence of a pancreatic pseudocyst leakage or ductal injury leading to leakage of pancreatic secretions into the peritoneum [1,2]. While the incidence of pancreatic ascites remains unknown, it is thought to occur more frequently in males between the ages of 20 and 50 [3]. Management is usually conservative, and includes holding oral feeds, providing parenteral nutrition, octreotide, and sometimes invasive procedures including endoscopic retrograde cholangiopancreatography (ERCP) and surgery [1,2]. Here, we present a rare case of ERCP induced pancreatic ascites with the aim to increase physician awareness of this condition.

## Case presentation

A 71- year-old man with medical history of cholelithiasis and cholecystectomy presented with increasing abdominal girth, weight loss, early satiety, and non-bloody, non-bilious vomiting ongoing for one month after undergoing endoscopic retrograde cholangiopancreatography for possible acute biliary pancreatitis at an outside hospital. ERCP records from the outside hospital revealed a benign-appearing distal CBD stricture. During that admission, the patient was managed conservatively, his symptoms resolved and he was then discharged. 

On review of symptoms, he denied any recent episodes of significant abdominal pain or recurrent abdominal pain. He denied any fever/chills, diarrhea/constipation, chest pain, shortness of breath, dysuria or hematuria. He also denied any recent changes to his medications.

On presentation, vitals were within normal limits. Labs were pertinent for: Sodium (Na)= 129 mEq/L (L) , Potassium (K)= 3.6 mEq/L (nl), Chloride (Cl)= 92 mEq/L (L), Albumin= <1.5 g/dL (L), Total bilirubin = 0.9 mg/dL (nl), Direct bilirubin = 0.4 mg/dL(nl), ALP= 235 U/L (H), GGT = 83 U/L (H), White blood cell count (WBC) = 11.4 K/uL (H), Hemoglobin (Hb) = 9.4 g/dL (L), Platelet (PLT) = 453 k/uL(H), Peritoneal fluid amylase = 9,635 IU/L (H), Peritoneal fluid total protein = 2.5g/dL (L), Peritoneal fluid LDH = 548 IU/L (H), Peritoneal fluid albumin = 0.8g/dL (L), Serum amylase = 38U/L (nl), Serum lipase =36U/L(nl). 

On physical examination, the patient appeared cachectic with a prominently distended abdomen, decreased bowel sounds and slight diffuse tenderness to palpation. 

**Figure 1:**
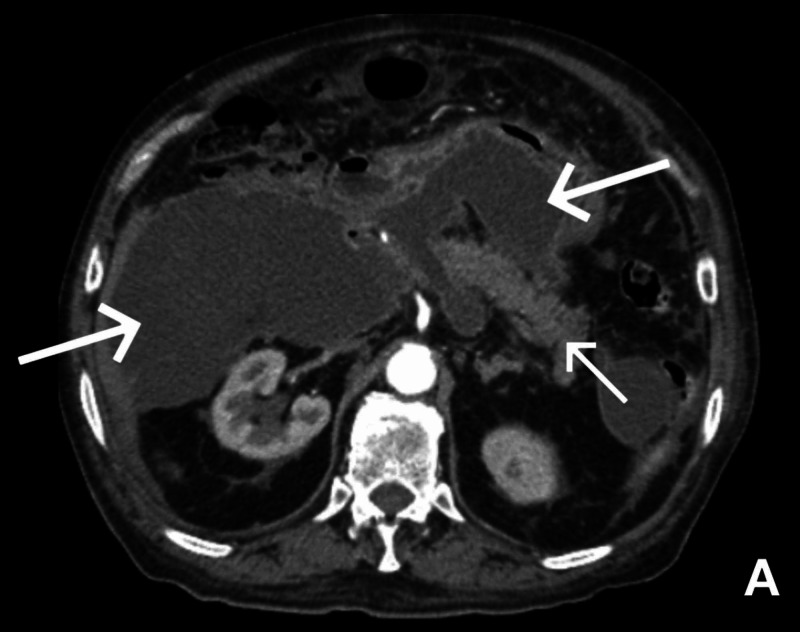
CT abdomen/pelvis with IV contrast and arrows showing multiple loculated fluid collections concerning for pancreatic pseudocysts vs abscesses. Smaller arrow is pointing to the pancreas while larger arrows are pointing to the fluid collections.

As part of initial workup, a CT abdomen was done and revealed loculated fluid collections concerning for pancreatic pseudocysts vs abscesses with associated massive ascites (Figure 1A)

The patient underwent drainage of the intrabdominal fluid collections. Retrieved fluid from the pseudocyst was cultured, and grew Citrobacter freundii, raising concern for an infected pancreatic pseudocyst. After drainage, a repeat CT showed slight interval resolution of the intraabdominal fluid collections. 

With concern for pancreatic ascites, the patient underwent magnetic resonance cholangiopancreatography (MRCP) to evaluate integrity of the pancreatic duct before ERCP. MRCP revealed an intact pancreatic duct without dilation and so ERCP was not attempted. 

The patient was managed conservatively with bowel rest, total parenteral nutrition, and serial paracentesis. Two weeks later, the team reintroduced enteral feeds via nasogastric tube in an effort to maintain intestinal integrity and prevent bacterial translocation. 

The patient’s hospital course was however, complicated by delirium, intractable vomiting, and refusal of serial paracentesis and enteral feeding. The patient was eventually transitioned home with hospice care. He was discharged with analgesics and antibiotic therapy for the infected pancreatic pseudocyst.

## Discussion

Pancreatic ascites describes accumulation of fluid into the peritoneum that occurs in the setting of pancreatic duct dehiscence, a ruptured pancreatic pseudocyst, or an abscess with walled off necrosis and fistula formation with leakage of pancreatic contents into the peritoneum [1-4]. Approximately 95% of reported pancreatic ascites cases have occurred in the setting of chronic pancreatitis. Albeit less frequently, pancreatic ascites have been reported in association with acute pancreatitis [5-8]. 

Due to the rarity of its presentation, the incidence, and presenting signs of pancreatic ascites are not well described in the literature. While literature remains scarce, some studies suggest the prevalence to be 3.5% in patients with chronic pancreatitis and 6-14% in patients with a pancreatic pseudocyst. Approximately, 10% of cases are idiopathic or of unknown cause [5, 6].

Typically, pancreatic ascites is exudative and characteristically presents with high fluid amylase levels of greater than 1000mg/dl. The protein level is usually greater than 3g/dL, and the serum ascites albumin gradient (SAAG) is usually less than 1.1g/dL [1,4].

The classic presentation of a patient with pancreatic ascites is slow, increasing abdominal girth, vague abdominal pain, and weight loss. Pleural effusions are unusually common and often cause exertional shortness of breath. Most patients with this condition also usually have a concomitant history of alcohol use and gall stone disease [4,7,8].

Due to its low prevalence, an evidence-based treatment modality is yet to be established for patients with pancreatic ascites. Sankaran et al. reported on the success of interventional therapy with ERCP, after conducting a retrospective study on pancreatic ascites patients in 1974 [8]. Chebli et al. in 2004 reported similar findings and recommended interventional therapy for patients with pancreatic ascites and major pancreatic duct disruption [9]. ERCP aids delineation of the ductal wall anatomy. Additionally, it can assist by reducing intraductal and pseudocyst pressures which drives destruction of the pancreatic duct, and causes pancreatic fluid leakage [4]. Despite the reported success of interventional therapy, other studies recommend conservative therapy. Conservative therapy includes bowel rest, providing nutrition via total parenteral nutrition, intermittent paracentesis, and somatostatin analogues (i.e. Octreotide). This modality is thought to allow for healing of pancreatic duct disruption and patient recovery. Gislason et al. reported a case of two patients that rapidly improved with infusion of a somatostatin analogue. Since then, another case report by Munshi et al. has also reported success with continuous infusion of octreotide which is a somatostatin analogue [10, 11].

Conservative management has been reported to have a failure rate of 40 to 60% with associated mortality rates of about 17% [3, 5, 12]. Comparatively, ERCP has been reported to have a failure rate of 14.3% and mortality rate of almost 0% [3, 13]. Since ERCP has been reported to have major success for management for pancreatic ascites, it is rarely the cause of pancreatic ascites. To date, only one other case report by Yunen et al. has recorded development of pancreatic ascites secondary to ERCP [14].

Other than the management options mentioned above, surgical therapy is another treatment modality used to manage pancreatic ascites. Generally, it is reserved for patients with pancreatic ascites due to trauma, and in patients resistant to conservative management. Surgical management is usually coupled with ERCP and has a reported a mortality rate of 15-25% [4].

As mentioned above, both chronic and acute pancreatitis have been described in literature as causes of pancreatic ductal injury and subsequent accumulation of peripancreatic fluid. While it is possible that our patient developed post ERCP pancreatitis and later a pancreatic pseudocyst with ascites, our patient denied having abdominal pain, nausea or vomiting post ERCP. Additionally, our patient’s lipase, which can remain elevated up to two weeks after pancreatitis, was normal. With this history, combined with the patient’s clinical presentation, we were inclined to believe ERCP induced ductal injury was a more likely cause of our patient’s ascites. 

## Conclusions

Pancreatic ascites describes a condition of fluid accumulation in the peritoneum that occurs in the setting of pancreatic duct injury and subsequent leakage of pancreatic fluid. While literature guiding management of pancreatic ascites is scarce, ERCP can be used diagnostically and therapeutically if done with endoscopic stenting. Compared to surgery and conservative management, ERCP has shown promise with reduced failure rates and mortality rates approaching 0%. Still, ERCP should be used with caution as the procedure itself can cause pancreatic injury and subsequent ascites as seen in our patient.To date, literature describing ERCP induced pancreatic ascites is infrequent. With this report, we aim to increase awareness in regards to ERCP as a potential cause of pancreatic ascites, and contribute to current literature by describing its clinical and radiographic presentation.

## References

[1] Gapp J, Holiat Gj, Chandra S (2020). Pancreatic ascites. S.

[2] Kanneganti K, Srikakarlapudi S, Acharya B, Sindhagatta V, Chilimuri S (2020). Successful management of pancreatic ascites with both conservative management and pancreatic duct stenting. Gastroenterology Res.

[3] Karlapudi S, Hinohara T, Clements J, Bakis G (2020). Therapeutic challenges of pancreatic ascites and the role of endoscopic pancreatic stenting. BMJ Case Rep.

[4] Gomez-Cerezo J, Barbado Cano A, Suarez I, Soto A, Rios JJ, Vasquez JJ (2020). Pancreatic ascites: study of therapeutic options by analysis of case reports and case series between the years 1975 and 2000. Am J Gastroenterol.

[5] Gupta S, Gaikwad N, Samarth A, Sawalakhe N, Sankalecha T (2020). Efficacy of pancreatic endotherapy in pancreatic ascites and pleural effusion. Med Sci (Basel).

[6] Kozarek RA (2007). Management of pancreatic ascites. Gastroenterol Hepatol (N Y).

[7] Cameron MD (2020). Chronic pancreatic ascites and pancreatic pleural effusions. Gastroenterology.

[8] Sankaran S, Walt A (2020). Pancreatic ascites: recognition and management. Arch Surg.

[9] Fonseca Chebli J, Gaburri P, Meirelles de Souza AF (2020). Internal pancreatic fistulas: proposal of a management algorithm based on a case series analysis. J Clin Gastroenterol.

[10] Munshi IA, Haworth R, Barie PS (2020). Resolution of refractory pancreatic ascites after continuous infusion of octreotide acetate. Int J Pancreatol.

[11] Gislason H, Gronbech JE, Soreide O (2020). Pancreatic ascites: treatment by continuous somatostatin infusion. Am J Gastroenterol.

[12] Lipsett PA, Cameron JL (2020). Internal pancreatic fistula. Am J Surg.

[13] Bracher GA, Manocha AP, DeBanto JR (2020). Endoscopic pancreatic duct stenting to treat pancreatic ascites. Gastrointest Endosc.

[14] Yunen RA, Goh KS, Chimbo-Osuagwu U, Azeez S (2012). Unusual case of pancreatic ascites and pancreatic pleuraleffusion following endoscopic retrogradecholangiopancreatography. IJCRI.

